# Diffusion Dynamics and Creative Destruction in a Simple Classical Model

**DOI:** 10.1111/meca.12085

**Published:** 2015-06-09

**Authors:** David Haas

**Affiliations:** ^1^ University of Graz

## Abstract

The article explores the impact of the diffusion of new methods of production on output and employment growth and income distribution within a Classical one‐sector framework. Disequilibrium paths are studied analytically and in terms of simulations. Diffusion by differential growth affects aggregate dynamics through several channels. The analysis reveals the non‐steady nature of economic change and shows that the adaptation pattern depends both on the innovation's factor‐saving bias and on the extent of the bias, which determines the strength of the selection pressure on non‐innovators. The typology of different cases developed shows various aspects of Schumpeter's concept of creative destruction.

## 1. INTRODUCTION

In this article, elements of the modern classical approach to economics and elements of evolutionary economics are combined to study the process of diffusion of a new and economically superior production method and its impact on output and employment growth and income distribution. This task is accomplished by adapting the Classical one‐commodity model of Kurz and Salvadori (1995, chap. 2) for the case in which several production methods are operated at the same time.

The study is inspired by two papers which explore the interface between the two schools of thought: Kurz (2008), who investigates the impact of different types of innovations on relative prices and wages using the long‐period method; and Metcalfe and Steedman (2013), who analyse the path connecting two long‐period positions (hereinafter LPP). Although the two papers differ in method and scope, both base their considerations on Schumpeter's (1934) vision of the process of technical change as a stylised sequence of invention, innovation and diffusion; and they agree that it is the process of diffusion which determines the economic effect of new ways of doing things. Further, both stress that the consequences for the system depend on the type of innovation. However, the above two papers emphasize different mechanisms that drive the process of adaptation: For Kurz (2008) as for Schumpeter (1934) imitation is key to adaptation, whereas Metcalfe and Steedman (2013) view differential growth as the prime mechanism of diffusion. The latter mechanism is central to the evolutionary account of economic selection and creative destruction. It causes adaptation through the change of the relative importance of innovators and non‐innovators within the population of firms and implies that investments are a necessary precondition for exploiting the economic potential of new methods of production (Silverberg and Verspagen, 2005).

The article adds to the literature on how the economy adapts by differential firm growth. The focus is on the question of how the type of innovation affects aggregate output growth, employment growth and income distribution. It is argued that innovations differ along two dimensions: The first dimension is the innovation's factor‐saving bias. The second dimension is the degree or extent of its bias, which is important because it determines the strength or intensity of the selection pressure on non‐innovators. In the article, we call the first dimension the *innovation bias* and the second one the *innovation intensity*. Both the implications of the innovation intensity and the impact of innovations on income distribution have not received much attention in the literature.

Adaptation paths are explored analytically and in terms of simulations. The study of disequilibrium paths is relevant for two reasons: First, understanding the dynamics outside equilibrium is crucial. Second, it adds to our understanding of how equilibria come about and it illuminates phenomena characterising the process of adaptation. The article shows that (1) the diffusion process drives aggregate performance via a number of effects which differ in direction and magnitude; (2) both the pattern of disequilibrium paths and the long‐term impact of the diffusion process depend on the bias and the intensity of the innovation; (3) the process of creative destruction depends on the characteristics of the innovation and under certain circumstances the destructive side of this process outweighs the creative one.

The article proceeds in three steps. Section 2 presents the basic relations and concepts needed to deal with multiple methods of production within a classical one‐sector framework and identifies the various channels through which diffusion affects aggregate growth. Based on this, a simple model of diffusion‐driven growth is specified in section 3. Section 4 explores the dynamics of the model and develops a typology of different cases allowing one to identify various forms and dimensions of Schumpeter's concept of creative destruction. Section 5 concludes.

## 2. HOW VARIETY AFFECTS GROWTH

The classical one‐sector growth model of Kurz and Salvadori (1995, chap. 2) is based on the following assumptions: In a one‐commodity world, a homogeneous good is produced by means of homogeneous labour and the good itself. Production functions are of the Leontief‐type and returns to scale are constant. Labour is available in abundance and wages are paid ex post. The good is taken as numéraire and the Law of One Price holds both with respect to the product and labour.

Variety is introduced into this framework by the assumption that in period *t*, *I* production methods contribute to total output. Method i∈I uses *a_i_* units of circulating capital and *l_i_* units of labour per unit of output. Given the uniform real wage rate *w_t_*, method *i* yields an ‘individual’ profit rate

(1) $$r_{i,t}=\frac{1-w_{t}l_{i}-a_{i}}{a_{i}}$$

Each production method *i* is identified with a firm, which produces $x_{i,t}$ units of output by employing $\kappa_{i,t}=a_{i}x_{i,t}$ units of productive capacity and $L_{i,t}=l_{i}x_{i,t}$ units of labour. Aggregate output is then given by $x_{t}=\sum_{i}x_{i,t}$, aggregate productive capacity by $\kappa_{t}=\sum_{i}\kappa_{i,t}$ and aggregate employment by $L_{t}=\sum_{i}L_{i,t}$. The market share of firm *i* in period *t* is given by $q_{i,t}=x_{i,t}/x_{t}$. Given the structure of production reflected by market shares, the average amount of capital and the average amount of labour needed to produce one unit of output are computed as

(2) $${\bar{a}}_{t}=\frac{\kappa_{t}}{x_{t}}=\sum_{i}q_{i,t}a_{i}$$

and

(3) $${\bar{l}}_{t}=\frac{L_{t}}{x_{t}}=\sum_{i}q_{i,t}l_{i}$$

The average production method $\left({\bar{a}}_{t},{\bar{l}}_{t}\right)$ reflects the average conditions of production at time *t* and thus is an abstract measure of the state of technical knowledge actually implemented. In addition, the *general rate of profit*

(4) $$r_{t}=\frac{\sum_{i}r_{i,t}\kappa_{i,t}}{\sum_{i}\kappa_{i,t}}=\frac{1-w_{t}{\bar{l}}_{t}-{\bar{a}}_{t}}{{\bar{a}}_{t}}$$

equals the profit rate of the average production method. Equation (4) further shows that the Classical inverse relationship holds between the general profit rate and the real wage rate for a *given* value of *q*.

Assume, that every firm ploughs back a fraction *s* of its profits into expansion of its own capacity such that firm *i*'s growth rate is given by

(5) $$g_{i,t}=\frac{x_{i,t+1}-x_{i,t}}{x_{i,t}}=sr_{i,t}$$

The investment propensity *s* is assumed to be uniform across firms. The growth rate of aggregate output then equals the weighted average of firm growth rates, where the weights are the firm market shares:

(6) $$g_{t}=\frac{x_{t+1}-x_{t}}{x_{t}}=s\sum_{i}q_{i,t}r_{i,t}=s{\bar{r}}_{t}$$

If individual rates of profit and thus firm growth rates differ, the structure of production changes over time. A little calculation shows that the market share of firm *i* evolves according to

(7) $$\frac{q_{i,t+1}-q_{i,t}}{q_{i,t}}=\frac{s\left(r_{i,t}-{\bar{r}}_{t}\right)}{1+s{\bar{r}}_{t}}$$

This implies that if the individual profit rate $r_{i,t}$ is larger (smaller) than ${\bar{r}}_{t}$, the market share of firm *i* increases (decreases). We refer to the gradual rise of some *q_i_* over time as the diffusion of production method *i* by *differential growth*.

A further relation helps dealing with the problem at hand. It is given by the following link between the evolution of total productive capacity and the general rate of profit:

(8) $$g_{\kappa ,t}=\frac{\kappa_{t+1}-\kappa_{t}}{\kappa_{t}}=s\sum_{i}\frac{\kappa_{i,t}}{\kappa_{t}}r_{i,t}=s\frac{1}{{\bar{a}}_{t}}\sum q_{i,t}\left(1-w_{t}l_{i}-a_{i}\right)=sr_{t}$$

As one can see by comparing equations (6) and (8), output growth depends on market share weighted average profit rate, while capacity accumulation depends on the general rate of profit. According to equation (2), the two growth rates do not necessarily coincide but are related by

(9) $$g_{t}=\frac{1+g_{\kappa ,t}}{1+\alpha_{t}}-1$$

This reveals that—given perfectly flexible labour supply—output growth has two sources: capacity growth, indicated by $g_{\kappa ,t}$, and capital biased technical change, indicated by rate of change $\alpha_{t}=\left({\bar{a}}_{t+1}-{\bar{a}}_{t}\right)/{\bar{a}}_{t}$. As both result from the process of investment, the two sources cannot be separated. Instead, the quantitative aspect of capacity accumulation and the qualitative change of the production structure appear as intertwined.

The above framework allows to identify different channels through which a diffusion process affects aggregate growth dynamics. A direct and an indirect channel can be distinguished. Both channels emanate from the mechanism of differential growth indicated by equation (7). The direct effect of diffusion is its impact on the average capital input coefficient. As one can see from equation (9), the output growth rate and the growth rate of the average capital coefficient are inversely related. We refer to that as the *technology effect* of diffusion. It may either be positive, negative or zero.

The indirect channel emanates from the fact that the diffusion process changes the average production method. In general, $\bar{a}$ and $\bar{l}$ will change such that at least one of the distributive variables increases. Any change of the general profit rate in turn affects aggregate capacity accumulation and hence output. Whether and to what extent this *general accumulation effect* is expansionary depends on the dynamics of the real wage rate. If the real wage adjusts, two complications emerge: First, if the real wage rate changes, all ‘individual’ profit rates change. If they change to different extents, the speed of structural change varies. Thus there is a feedback between the dynamics of the real wage rate and the process of diffusion. We call it the *wage feedback effect*. Second, if the real wage rate increases such that some of the ‘individual’ profit rates turn negative, some firms are forced to decline or even to exit. This *differential accumulation effect* provides a further indirect channel, which dampens capacity accumulation and hence output growth.

Because of the multiple effects and complications involved, the consequences of a change of the production structure for aggregate dynamics are not obvious *a priori*. As some effects might accelerate whereas others might decelerate growth, it is not even clear whether the diffusion process speeds up or slows down growth. Rather, the implications depend (1) on the bias and intensity of the innovation, (2) on the behavioural responses of firms, and (3) on the dynamics of the real wage rate.

To shed some light on the relation between a change in the production structure and aggregate dynamics, the remaining parts of the article presents and analyses a simple model of diffusion‐driven growth in which we consider only two methods of production and only some of the above effects.^1^ The focus is on the implications of the innovation bias and intensity for the dynamics of output, employment and income distribution. Both transitional effects and long‐term impacts on output and employment levels are explored. A typology of cases for different kinds of innovations is developed and allows us to identify the various forms and dimensions of Schumpeter's concept of creative destruction involved in the restructuring process.

## 3. A SIMPLE MODEL OF DIFFUSION‐DRIVEN GROWTH

For analytical convenience the following analysis concentrates on the stylized case of two rival methods of production; further, the problem of how the innovation emerges is set aside as both methods are assumed to be already in place.

Let method 1 denote the established one and method 2 the innovation. The two ‘individual’ profit rates are given by equation (1). The output share of method 2 is given by $q_{t}=x_{2,t}/x_{t}$ and the market share of the incumbent method 1 by $1-q_{t}=x_{1,t}/x_{t}$. The two central assumptions the model is based on concern a simple wage setting rule (section 3.1) and a refined version of the investment function (5), which includes the case of negative individual profit rates (section 3.2).

### 3.1 Wage dynamics

As already mentioned, the Classical inverse relationship holds between the general profit rate and the real wage rate for a *given* value of *q*. But since the superior method 2 allows for a higher surplus of production, there is the question of how this additional surplus is distributed among capitalists and workers when *q* increases.^2^

We impose a wage adjustment rule which determines the wage rate endogenously but keeps things as simple as possible. It is assumed that the real wage rate *w* adjusts to a change in *q* by taking the general profit rate as constant and exogenously given. As *r_t_* = *r*, equation (4) turns into the wage setting rule

(10) $$w_{t}=\frac{1-(1+r){\bar{a}}_{t}}{{\bar{l}}_{t}}$$

This wage setting rule has some ‘Classical’ flavour, since one of the two distributive parameters is assumed to be constant and exogenously given, whereas the other is determined by the conditions of production, which are changing in our case. It may be motivated by the empirical observation that the general profit rate is a trend‐less magnitude. However, this assumption implies that the diffusion process does not influence aggregate growth via the *general accumulation effect*. Attention therefore focuses on the remaining channels which are put in sharp relief.

From the wage setting rule (10) it follows that the real wage rate is bound to rise during diffusion: In the LPP before the innovative method has entered the system (*q* = 0), the wage rate is determined by method of production 1 only, which is characterised by $\left(a_{1},l_{1}\right)$ and an ‘individual’ profit rate $r_{1}=r$. If *q* = 1, production method 2 just obtains a profit rate $r_{2}=r$ and the corresponding wage rate is determined by method of production 2 only. If *q* gradually rises from 0 to 1, $\bar{a}$ and $\bar{l}$ change such that the real wage rate increases via equation (10). This in turn reduces all ‘individual’ rates of profit via equation (1) but leaves unchanged the general rate of profit.

As the general rate of profit is constant, define the *rate of extra profits* of method *i* as $\rho_{i}=r_{i}-r$, where

(11) $$\rho_{i}=\frac{1-wl_{i}-\left(1+r\right)a_{i}}{a_{i}}$$

By comparing *r_i_* and *r*, the relative superiority of some method of production is specified. Methods of production which yield positive (negative) extra profits have a cost advantage (disadvantage) compared to the average conditions of production and are economically superior (inferior).

An important implication of the proposed definitions of the general rate of profit and of the rate of extra profits, given by equations (4) and (11), respectively, is that in each period *total extra profits* sum up to zero:

(12) $$(1-q_{t})\rho_{1,t}a_{1}+q_{t}\rho_{2,t}a_{2}=0$$

This relation is central for the analysis of the dynamics of growth and income distribution (see section 4).

### 3.2 Investment behaviour

We implement a simple form of retained earning dynamics based on the following assumptions: (1) Investment behaviour is uniform across firms.^3^ (2) If firm *i* invests, it ploughs back part of its profits into building capacity *i*. Although firm investment does not adapt to differential profitability, over time the fraction of total investment which flows into capacity expansion of the innovative process increases due to differential growth. (3) Firms operate at *full capacity* throughout. As we only consider circulating capital, productive capacity of *i* is determined by last period's investments into *i*.

According to equation (11), the output of a firm using *i* at time *t*, $x_{i,t}$, is the sum of wage payments, capital investment to maintain the output‐level and profits:

$$x_{i,t}=w_{t}l_{i}x_{i,t}+a_{i}x_{i,t}+(r+\rho_{i,t})a_{i}x_{i,t}$$

To determine next period's output produced by method of production *i*, $x_{i,t+1}$, the following variation of the classical investment hypothesis formulated at the level of firms is adopted: Let s∈(0,1] be the propensity to invest in case of a positive rate of profit $r+\rho_{i,t}>0$ and let $C_{i,t}\geq 0$ denote consumption out of profits; there are no savings out of wages. Three cases can be distinguished:

***Case 1***: $r+\rho_{i,t}>0$. If the firm yields a positive rate of profit it has the potential to grow. After paying wages, the amount of investible resources $x_{i,t}-wl_{i}x_{i,t}$ splits up into replacement investment $a_{i}x_{i,t}$, net investment $s(r+\rho_{i,t})a_{i}x_{i,t}$ and capitalist consumption $(1-s)\left(r+\rho_{i,t}\right)a_{i}x_{i,t}$. As net investment is positive, the firm accumulates and its output increases. Hence, $C_{i,t}>0$ and $x_{i,t+1}>x_{i,t}$.
***Case 2***: $-1<r+\rho_{i,t}\leq 0$. In this case net investment is negative because the amount of investible resources $x_{i,t}-wl_{i}x_{i,t}$ is smaller than replacement investments $a_{i}x_{i,t}$. As capitalist consumption is non‐negative, the firm devotes the whole amount of its investible resources $(1+r+\rho_{i,t})a_{i}x_{i,t}$ to capacity construction. This implies that $C_{i,t}=0$ and that $x_{i,t+1}\leq x_{i,t}$.
***Case 3***: $r+\rho_{i,t}\leq -1$. Since now $wl_{i}x_{i,t}\geq x_{i,t}$, the firm using production method *i* is just able to pay for the total wage bill or even fails to pay for it. As no resources remain with the firm, it is no longer able to continue its business but is forced to exit. In this case the firm pays its workers what it can and then leaves the market. Hence, $x_{i,t+1}=0$.

Summing up, output growth of firm *i* is given by

(13) $$g_{i,t}=\frac{x_{i,t+1}-x_{i,t}}{x_{i,t}}=\{\begin{array}{ll} s\;(r+\rho_{i,t}) & \text{in}\;\text{Case}\;1:\;r+\rho_{i,t}>0\\ r+\rho_{i,t} & \text{in}\;\text{Case}\;2:\;-1<r+\rho_{i,t}\leq 0\\ -1 & \text{in}\;\text{Case}\;3:\;r+\rho_{i,t}\leq -1 \end{array}$$

**Figure 1:**
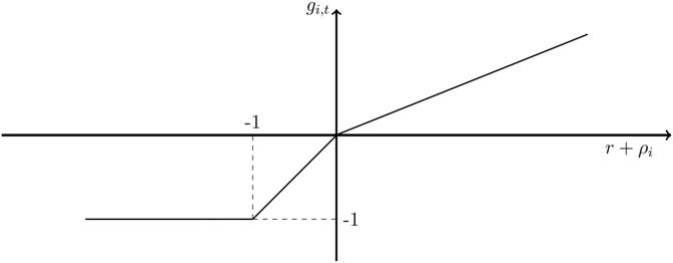
Kinked investment function for s = 0.4.

The investment function is illustrated in figure 1. Comparing the three cases shows an asymmetry between a firm's growth and decline. This asymmetry reflects a basic difference between a firm that reaps profits and a firm that incurs losses. On the one hand, a positive rate of profit implies a potential to grow and allows the firm to *decide* on how much to expand its capacity. This decision is reflected by its propensity to invest. Conversely, a negative rate of profit implies a loss and *enforces* a firm to reduce its capacity.

Moreover, a firm facing a loss is confronted with the question of whether to continue production or not. Instead of the conventional argument that the firm stops producing and shuts down as soon as the price falls below the average variable costs (i.e. when $r+\rho_{i,t}\leq 0$), we assume that firms stay in business as long as they can. This assumption relies on arguments put forth by Kahn (1989 [1929]), who argues that a firm facing a loss is likely to stay in business to preserve its operational capability and business connections which otherwise would be lost. Further, (psychological) restrictions may retard fundamental changes of firm policies in the short run. However, the ability to continue production at a loss ultimately exhausts. This is reflected in Cases 2 and 3 of the investment hypothesis: Firms decide to maintain production and continue business at a loss. Their output declines via a disinvestment process and they gradually run out of means of production. While a firm in the second case still is able to continue production at a smaller scale, the firm in the third case ceases to be able to maintain business.

**Figure 2:**
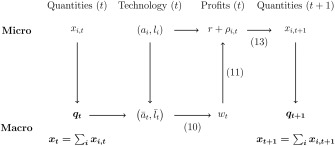
Logic of the model.

Figure 2 illustrates the logic of the model. At the beginning of each period *t*, production starts. Given $x_{i,t}$, *q_t_* is computed. Given the market share, the average conditions of production are computed. In the second step, the surplus is distributed and the real wage rate *w_t_* is determined by equation (10). Given the technical coefficients *a_i_* and *l_i_*, individual profit rates $r+\rho_{i,t}$ are determined as residuals by equation (11). Given profit rates, investment is determined by equation (13) which is the basis for production in period *t* + 1.

## 4. FORMS AND DIMENSIONS OF CREATIVE DESTRUCTION

In this section, we adopt the model presented above to analyse how the diffusion process shapes aggregate dynamics. The effects involved in the restructuring process and its consequences are shown to depend on the type of innovation. Overall, the analysis reveals that the process of *creative destruction* (Schumpeter 2010 [1942], chap. VII), the replacement of old methods of production by new ones, manifests itself in different forms and dimensions.

Section 4.1 present a typology of innovations along two dimensions, bias and intensity. The dynamics of aggregate growth are analysed in section 4.2 and the pattern of diffusion in section 4.3. In sections 4.4 and 4.5 the implications for employment and the wage share for different types of innovations is discussed.

### 4.1 Bias and intensity of innovations

In the following, we use the concept of the factor space to define and illustrate different types of innovations. Basically, any invading method 2 is characterised by its *innovation bias* reflecting its factor saving characteristics with respect to the incumbent method of production 1 and by the degree of its bias, which determines the intensity of the selection pressure on the incumbent firm. The innovation bias depends on the sign of the relative change of the capital and labour input coefficients

$$\Theta_{a}=\frac{a_{2}-a_{1}}{a_{1}}\geq -1\;\text{and}\;\Theta_{l}=\frac{l_{2}-l_{1}}{l_{1}}\geq -1$$

and the innovation intensity relates to the magnitude of these two measures.

**Figure 3:**
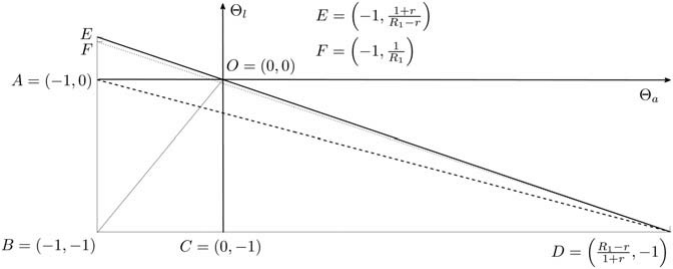
Partition of the factor space for $R_{1}=4$ and r = 0.1.

The two measures Θ*_a_* and Θ*_l_* are the axes of the factor space, which is illustrated in figure 3 for a given general rate of profit *r* = 0.1 and a given maximum rate of profit of method 1, $R_{1}=\left(1-a_{1}\right)/a_{1}=4$. The incumbent method of production is located at the origin. The line $\bar{BCD}$ is characterised by $\Theta_{l}=-1$ (i.e. by $l_{2}=0$) and the line $\bar{BAE}$ is characterised by $\Theta_{a}=-1$ (i.e. by $a_{2}=0$). The downward sloping *iso‐profit rate line*

$$\bar{DE}:\;\Theta_{l}=-\frac{1+r}{R_{1}-r}\Theta_{a}$$

defines the set of all methods of production which have the same unit costs of production as the incumbent method at the ruling wage rate. Hence, methods of production lying within the triangle ΔDEB are potential innovations, since they permeate the system if introduced. Methods of production lying above $\bar{DE}$ are inferior compared to the incumbent method and do not succeed if introduced. They can be termed inventions that will not become innovations.^4^ The set of potential innovations given by the triangle ΔDEB can be partitioned according to the innovation bias. Table 1 provides a list with all possible types of innovation biases.

**Table 1:** Different forms of innovation biases.

Innovation Bias	Technical Coefficients	In Figure 3
Capital saving and labour using	$\Theta_{a}<0,\;\Theta_{l}>0$	ΔOEA
Labour saving and capital using	$\Theta_{a}>0,\;\Theta_{l}<0$	ΔOCD
Pure capital saving	$\Theta_{a}<0,\;\Theta_{l}=0$	$\bar{OA}$
Pure labour saving	$\Theta_{a}=0,\;\Theta_{l}<0$	$\bar{OC}$
Combined factor saving	$\Theta_{a}<0,\;\Theta_{l}<0$	ΔOABC
Neutral	$\Theta_{a}=\Theta_{l}<0$	$\bar{OB}$
Dominantly capital saving	$\Theta_{a}<\Theta_{l}<0$	ΔOAB
Dominantly labour saving	$0>\Theta_{a}>\Theta_{l}$	ΔOBC

The magnitude of the two biases determines the innovation intensity. Three types thereof can be distinguished according to what happens to the ‘individual’ rate of profit of the incumbent method after the diffusion process is accomplished. The line

$$\bar{DF}:\;\Theta_{l}=-\frac{r}{R_{1}}-\frac{1+r}{R_{1}}\Theta_{a}$$

defines the set of all innovations for which the incumbent method generates exactly zero total profits in the *new* LPP: $r+\rho_{1}{|}_{q=1}=0$. And the line

$$\bar{AD}:\;\Theta_{l}=-\frac{1+r}{1+R_{1}}(1+\Theta_{a})$$

defines the set of all innovations for which the incumbent method is just able to pay the total wage bill in the *new* LPP: $r+\rho_{1}{|}_{q=1}=-1$. These two lines classify potential innovations into three types of innovation intensity differing with respect to intensity of selection pressure on the incumbent firm and its ability to survive. The ultimate fate of the incumbent firm is one central dimension of the process of *creative destruction* which takes one of three forms:

#### 4.1.1 Low selection pressure

If the invading method lies within the triangle ΔDEF in figure 3, the incumbent firm yields a positive rate of profit in the new LPP. It follows that it is neither forced to decline in absolute terms nor forced to exit. It rather co‐exists with the innovative firm even in the long run.

#### 4.1.2 Medium selection pressure

If the invading method lies within the triangle ΔDFA, the ‘individual’ rate of profit of the incumbent firm lies between −1 and 0 in the new LPP. It is still able to continue business but it asymptotically declines in absolute terms due to losses. Thus only the innovative firm survives as time approaches infinity.

#### 4.1.3 High selection pressure

Any invading method lying within the triangle ΔDAB implies an ‘individual’ rate of profit smaller than −1 for the incumbent method in the new LPP. Thus the incumbent firm is forced to exit in finite time; firm exit is the strongest evidence of creative destruction in this setting.

Note that the above comparative analysis is independent of the proposed wage adjustment rule but solely relies on the assumption that the diffusion process has no lasting effect on the general rate of profit. Yet, the intensity of some innovation and thus the incumbent firm's ability to survive depends on the general rate of profit: A high general rate of profit tends to protect the incumbent firm against the more severe consequences of decline and exit as the wedge representing innovations of low intensity ΔDEF is bigger for a higher *r*. However, this wedge is very narrow for reasonable values of *r*. Thus the cases of medium and high selection pressures or intensity are decisive. In the following analysis we focus on the case of medium intensity and abstract from the latter cases; in this way we keep the number of firms constant.

### 4.2 Aggregate growth

In this section, we explore how innovation bias and innovation intensity shape the aggregate output growth rate. Because of equations (13) and (6), the growth rate of aggregate output is given by

(14) $$g_{t}=\{\begin{array}{ll} s\;(r+{\bar{\rho }}_{t}) & \text{in}\;\text{Case}\;1:\;r+\rho_{1,t}>0\\ s\;(r+{\bar{\rho }}_{t})+(1-s)(r+\rho_{1,t})(1-q_{t}) & \text{in}\;\text{Case}\;2:\;-1<r+\rho_{1,t}\leq 0\\ s\;r & \text{in}\;\text{Case}\;3:\;r+\rho_{1,t}\leq -1 \end{array}$$

where ${\bar{\rho }}_{t}=\left(1-q_{t}\right)\rho_{1,t}+q_{t}\rho_{2,t}$ denotes the market share weighted average rate of extra profits, and the growth rate of aggregate productive capacity by

$$g_{\kappa ,t}=\{\begin{array}{ll} s\;r & \text{in}\;\text{Case}\;1:\;r+\rho_{1,t}>0\\ \left(1-q_{t})\frac{a_{1}}{{\bar{a}}_{t}}(r+\rho_{1,t})+q_{t}\frac{a_{2}}{{\bar{a}}_{t}}s(r+\rho_{2,t}\right) & \text{in}\;\text{Case}\;2:\;-1<r+\rho_{1,t}\leq 0\\ s\;r & \text{in}\;\text{Case}\;3:\;r+\rho_{1,t}\leq -1 \end{array}$$

These two equations form the basis for the analysis of how the diffusion process affects output growth. Because of the proposed wage setting rule the general rate of profit *r* is constant and therefore the general accumulation effect does not affect aggregate growth. The two effects we focus on are the *technology effect* and the *differential accumulation effect*. They are analysed in the following.

#### 4.2.1 Technology effect

Consider the extreme case *s* = 1, in which the kink in the investment function and thus the asymmetry between firm growth and decline vanishes. The aggregate growth rate is then given by $g_{t}=r+{\bar{\rho }}_{t}$. From equation (12) it follows that

(15) $${\bar{\rho }}_{t}=(1-q_{t})\rho_{1,t}+q_{t}\rho_{2,t}=-q_{t}\rho_{2,t}\Theta_{a}.$$

It furthermore holds that *q_t_* and $\rho_{2,t}$ are strictly positive implying that the sign of ${\bar{\rho }}_{t}$ is the negative of the sign of Θ*_a_*. Thus three cases can be distinguished:

1. ${\bar{\rho }}_{t}<0$ in the case of labour saving and capital using innovations ( $\Theta_{a}>0$);
2. ${\bar{\rho }}_{t}=0$ in the case of pure labour saving innovations ( $\Theta_{a}=0$);
3. ${\bar{\rho }}_{t}>0$ in the case of capital saving innovations ( $\Theta_{a}<0$).

**Figure 4:**
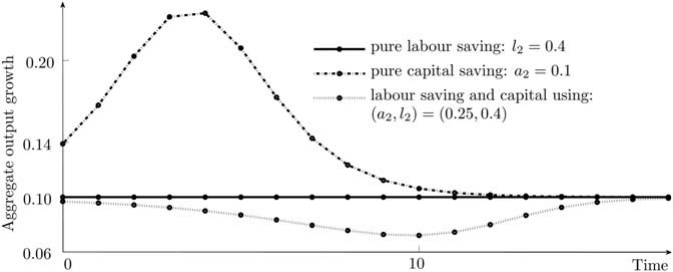
Technology effect for $\left(a_{1},l_{1})=(0.2,0.5\right)$ and r = 0.1.

Figure 4 illustrates the three possible patterns arising due to the technology effect. Whereas the diffusion of the pure capital saving method accelerates aggregate growth, labour saving and capital using technical change slows down economic growth. Only the diffusion of the pure labour saving method does not affect output growth via this channel.^5^

#### 4.2.2 Differential accumulation effect

Let us now turn to the implications of the asymmetry between firm growth and decline reflected by the kinked investment function (13). This effect depends on the innovation intensity and dampens aggregate growth via decelerating aggregate capacity accumulation. If the invading method lies within the area ΔDAF, the real wage rate lies above the maximum wage rate process 1 can pay without making losses, given by ${\hat{w}}_{1}=(1-a_{1})/l_{1}$, for all $q_{t}\in (q_{0},1]$.^6^ The respective threshold market share *q*_0_ is determined by

$$q_{0}=\frac{-r}{(1+r)\Theta_{a}+R_{1}\Theta_{l}}$$

**Figure 5:**
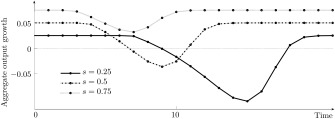
Differential accumulation effect for $\left(a_{1},l_{1})=(0.2,0.5\right)$ and $\left(a_{2},l_{2})=(0.2,0.4\right)$, with r = 0.1.

For *s* < 1 and $q_{t}\in \left(q_{0},1\right]$ the differential accumulation effect influences aggregate growth. This is illustrated in figure 5 for different investment propensities *s* and for the case of a pure labour saving innovation, which shows no technology effect. The value of *q*_0_ is negatively correlated with the propensity to invest and the differential accumulation effect is stronger for smaller values of *s*.

#### 4.2.3 Interaction of effects

**Figure 6:**
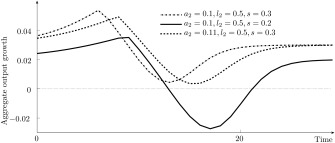
Interaction of the technology and the differential accumulation effect for $\left(a_{1},l_{1})=(0.2,0.5\right)$ and r = 0.1.

The diffusion of a new method of production with $\Theta_{a}<0$ small enough to turn the profit rate of the inferior method negative at some *q*_0_ leads to a wave‐like path of the aggregate growth rate for the following reasons: Initially all firms experience a positive rate of profit and the technology effect accelerates growth. Yet, as soon as the profit rate of firms using the old method turns negative, aggregate growth is dampened due to the differential accumulation effect. Figure 6 provides an illustration for different examples of pure capital saving innovations.

#### 4.2.4 Long‐term impact

To assess the long‐term impact of the diffusion process on growth, the non‐steady growth path is compared with the steady‐state output path defined by *q_t_* = 0. In this latter *business‐as‐usual* (BAU) scenario the innovation is not introduced and output at time *T* is given by ${\hat{x}}_{T}={\left(1+s\hat{r}\right)}^{T}x_{0}$ with $\hat{r}=r$ for some initial output *x*_0_ and propensity to save *s*. The two output levels at time *T* > 0 are related by

$$\Delta_{s}(T)=\frac{x_{T}}{{\hat{x}}_{T}}=\prod_{t=1}^{T}\frac{1+g_{t}}{1+sr}$$

**Figure 7:**
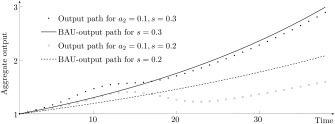
Long‐term effect on the output level for $\left(a_{1},l_{1})=(0.2,0.5\right)$ and r = 0.1.

This product series provides an assessment of the overall long‐term impact of diffusion‐driven growth. For the first two examples of figure 6 with $a_{2}=0.2$, one gets $\Delta_{0.3}(38)=0.967$ and $\Delta_{0.2}(38)=0.768$. Thus, for *s* = 0.3 (*s* = 0.2) the long‐term output is 3.3% ( 23.2%) smaller than BAU output. How these two output paths compare with their respective BAU paths is illustrated in figure 7.

The analysis and numerical examples show that although the diffusion process does not affect the LPP growth rate, short‐term fluctuations have long‐term implications on the output level. Moreover, the differential accumulation effect may outweigh the technology effect. If they are working in opposite direction, the economy may get on a path on which the output level is persistently below the BAU output.

Summing up, non‐constant aggregate growth is a further aspect of creative destruction. The effects shaping the time profile of aggregate output depend on the bias and the intensity of the invading innovation. As the gradual replacement process involves both growth‐enhancing and growth‐depressing effects, the growth regime may flip during the process and the destructive impact may well predominate the creative one.

### 4.3 Diffusion pattern

Based on the above discussion, this section explores how the two dimensions characterising an innovation shape the pattern of diffusion. As the growth rate of the innovating firm is given by equation (13) and the evolution of total output is given by equation (14), the market share $q_{t}=x_{2,t}/x_{t}$ of the innovation evolves according to

(16) $$\frac{q_{t+1}}{q_{t}}=\{\begin{array}{ll} \frac{1+s(r+\rho_{2,t})}{1+s(r+{\bar{\rho }}_{t})} & \text{in}\;\text{Case}\;1:\;r+\rho_{1,t}>0\\ \frac{1+s(r+\rho_{2,t})}{1+s(r+{\bar{\rho }}_{t})+(1-s)(r+\rho_{1,t})(1-q_{t})} & \text{in}\;\text{Case}\;2:\;-1<r+\rho_{1,t}\leq 0\\ 1 & \text{in}\;\text{Case}\;3:\;r+\rho_{1,t}\leq -1 \end{array}$$

Generally, the mechanism of differential growth generates a sigmoid diffusion pattern and mimics the stylised fact of S‐shaped curves of diffusion processes (Stoneman, 2002). But they to not resemble a simple logistic curve, because total output evolves at a non‐constant rate (Metcalfe and Steedman, 2013).

Additional complications arise if the wage rate adapts endogenously: The rise of the real wage rate reduces the (extra) profit rates to different extents if the two methods of production differ with respect to their capital intensity $l_{i}/a_{i}$. To see this, equation (16) for Case 1 can be rewritten as follows:

$$\frac{q_{t+1}-q_{t}}{q_{t}}=\frac{1+s\left(1-q_{t}\right)\Delta \rho_{t}}{1+s(r+{\bar{\rho }}_{t})}$$

where $\Delta \rho_{t}=\rho_{2,t}-\rho_{1,t}>0$ denotes the profit rate differential, given by

$$\Delta \rho_{t}=\left(\frac{1}{a_{2}}-\frac{1}{a_{1}}\right)-w_{t}\left(\frac{l_{2}}{a_{2}}-\frac{l_{1}}{a_{1}}\right)$$

It follows that if the innovation is capital saving and labour using, the increase of the wage rate decelerates the diffusion speed. But if the innovation is capital using and labour saving, the increase of the wage rate accelerates the diffusion speed. Only in the case of a neutral innovation, this *wage feedback effect* does not alter the speed of adaptation. Note that although the speed of diffusion might be affected, the direction at which market shares change does not vary in the case of two methods of production. In addition, also the intensity of the innovation bias determines the magnitude of the profit rate differential $\Delta \rho_{t}$ and thus the speed of diffusion.

The speed of the diffusion process and its overall pattern provides a further element involved in the process of creative destruction, which depends on the bias and intensity of the innovation and takes different forms: (1) an asymptotic diffusion path due to absolute growth but *relative decline* of incumbent firms in the case of a low innovation intensity; (2) an asymptotic diffusion path accelerated by the *absolute decline* of incumbent firms in the case of medium innovation intensity. This can be seen by looking at the negative term $\left(1-s)(r+\rho_{1,t})(1-q_{t}\right)$ in the denominator of Case 2 which indicates the impact of the incumbent firm's absolute decline; (3) in the case of high intensity the diffusion process is accomplished in finite time by the extinction of the incumbent method of production.

### 4.4 Employment growth

This section explores how the innovation bias and its intensity affect the evolution of total employment $L=L_{1}+L_{2}$. Given equations (2) and (3), aggregate employment evolves according to

(17) $$g_{L,t}=\frac{L_{t+1}-L_{t}}{L_{t}}=\left(1+g_{\kappa ,t}\right)\frac{\left(1+\lambda_{t}\right)}{\left(1+\alpha_{t}\right)}-1$$

where $\lambda_{t}=\left({\bar{l}}_{t+1}-{\bar{l}}_{t}\right)/{\bar{l}}_{t}$ is the rate at which the average labour coefficient changes. From equation (17) it follows that employment growth is influenced by the innovation intensity via the differential accumulation effect and the innovation bias.

Restricting our attention to the latter, let

$$\Gamma_{t}=\frac{\left(1+\lambda_{t}\right)}{\left(1+\alpha_{t}\right)}=\frac{1+q_{t}\Theta_{a}}{1+q_{t+1}\Theta_{a}}\frac{1+q_{t+1}\Theta_{l}}{1+q_{t}\Theta_{l}}$$

As $q_{t+1}>q_{t}$ and $\Theta_{a},\Theta_{l}\geq -1$, three cases can be distinguished:

1. $0<\Gamma_{t}<1$ in cases for which $\Theta_{a}>\Theta_{l}$, e.g. in the case of a dominantly labour saving innovation.
2. $\Gamma_{t}=1$ in the case of a neutral innovation ( $\Theta_{a}=\Theta_{l}$).
3. $\Gamma_{t}>1$ in the case for which $\Theta_{a}<\Theta_{l}$, e.g. in the case of a dominantly capital saving innovation.

This list indicates job creation and destruction as another aspect of the process of creative destruction. For example, the diffusion of a pure labour saving innovation of low intensity decelerates the job creation rate such that the resulting employment path lies below its BAU employment path. If a dominantly labour saving innovation of medium intensity diffuses, the incumbent firm's absolute decline results in the destruction of jobs, which outweighs the creation of new jobs due to the differential accumulation effect. Again, as some new jobs are created and some old jobs are destroyed at the same time, the overall long‐term impact of the diffusion process on the employment level may be negative.

### 4.5 Income distribution

In this section, we explore the change of the income shares due to the diffusion of a process innovation. The wage share *ω_t_* is defined as $\omega_{t}=W_{t}/\left(W_{t}+P_{t}\right)$ with *W_t_* denoting total wage payments and *P_t_* total profits at time *t*. It evolves according to

(18) $$\omega_{t}=\frac{w_{t}{\bar{l}}_{t}x_{t}}{w_{t}{\bar{l}}_{t}x_{t}+r{\bar{a}}_{t}x_{t}}=1-\frac{r}{R_{t}}$$

where $R_{t}=(1-{\bar{a}}_{t})/{\bar{a}}_{t}$ denotes the maximum rate of profits of the average production method.

Equation (18) reveals two basic channels through which the diffusion process affects the wage share: To begin with, there is an inverse relationship between the wage share and the general rate of profit, given *R_t_*. This is due to the fact that the *sum* of total extra profits is zero throughout (see equation (12)); even if the average *rate* of extra profits is non‐zero, differential ‘individual’ extra profits imply a re‐distribution of income within the group of capitalists without affecting income shares. The direction of change of the wage share therefore is closely related to the dynamics of the real wage rate.

In the case assumed here, the general rate of profit *r* is constant. The diffusion process therefore affects the direction of change of the wage share only via its impact on the maximum rate of profits of the average production method. This measure in turn depends on the innovation bias.

To see how it affects the wage share, let $R_{i}=(1-a_{i})/a_{i}$ denote the maximum rate of profits of production method *i*. The wage share in the LPP with method of production (*a_i_*, *l_i_*) being used then is given by

$$\omega_{i}=1-\frac{r}{R_{i}}$$

A comparison of the wage share before the innovative process enters the system (*ω*_1_) with the wage share which prevails after the diffusion is accomplished (*ω*_2_) shows the following:

1. If the innovation is capital using ( $\Theta_{a}>0$), $R_{2}<R_{1}$ and the wage share falls: $\omega_{2}<\omega_{1}$.
2. If the innovation is pure labour saving ( $\Theta_{a}=0$), *R*_2_ = *R*_1_ and the wage share does not change: $\omega_{2}=\omega_{1}$.
3. If the innovation is capital saving ( $\Theta_{a}<0$), $R_{2}>R_{1}$ and the wage share increases: $\omega_{2}>\omega_{1}$.

Because the difference between the two maximum rates of profit is given by $R_{2}-R_{1}=-\Theta_{a}/a_{2}$, there is a symmetry between the technology effect on growth and the change in the wage share: Pure labour saving technical change neither affects aggregate growth nor does it affect the income shares, whereas capital using technical change dampens aggregate growth and reduces the wage share. All other forms of technical change increase both aggregate growth and the wage share. But, while the technology effect is related to the average rate of extra profits, the effect on income distribution arises from the change of the maximum rate of profit.

## 5. CONCLUSIONS

The article has discussed the consequences of a changing structure of production for firms and for the economy as a whole. The analysis uses a simple one‐commodity framework in which two methods of productions initially co‐exist. Adjustment of the structure of production is brought about by differential growth of firms. This mechanism implies that economically superior methods of production gradually supersede inferior ones.

Our study shows that economic development proceeds in a non‐constant way and involves a number of effects that differ in direction and magnitude. It is demonstrated that the diffusion of new methods of production need not always result in higher output and employment growth. Rather, the overall impact on economic performance depends on the relative strength of counteracting tendencies, which can be traced back to the type of innovation. Both the innovation bias and intensity are major determinants of the process that unfolds after an innovation has upset the system. The typology of different cases developed reveals that the process of creative destruction involves different forms and dimensions. These findings suggest that a steady‐state analysis is not sufficient to fully grasp the mechanisms and consequences involved in the process of technical change.

There are two issues that deserve further attention: First, firms are assumed not to respond to profit differentials: neither do they try to imitate nor to adjust their investment flows. How the above results change if firm behaviour is assumed to be less inertial is a topic for further investigation. Second, by assuming a given and constant general rate of profit one effect on aggregate dynamics of the diffusion process is excluded from the analysis. An exploration of the general accumulation effect is linked to the question of how labour market conditions interact with the process of diffusion. For a study along this line the wage adjustment mechanism proposed may serve as a benchmark.

## Footnotes

1 Also the problems of credit creation and of the non‐neutrality of money, which are central elements in Schumpeter's analysis of innovation and adaptation, are set aside in this article; see Caiani et al. (2014) who trace the interdependencies between the ‘real’ process of structural change and financial dynamics within a ‘monetary theory of production’ framework.

2 In the literature on diffusion dynamics different wage setting rules can be found: In Nelson (1968) the uniform wage rate is pushed up by the increase of the profit share. Nelson and Winter (1982, chap. 10) impose a neoclassical upward‐sloping labour supply curve. In Silverberg (1984) the real wage rate rises as the employment ratio increases. Englmann (1992) compares the implications of different wage adjustment rules for employment in a simulation study. The case of wage dispersion is explored by Nelson and Pack (1999) and Metcalfe and Steedman (2013), who assume that innovators pay a fixed wage premium. Apart from the latter, labour is assumed to be in flexible supply in this literature; the view that the availability of labour is not a binding constraint to accumulation is also a distinguishing feature of the classical approach to growth and distribution (Michl, 2000).

3 See Metcalfe (2012) for an analysis of how diffusion proceeds if firms differ both in their profitability and in their investment behaviour.

4 Whether some method of production is a potential innovation or not depends on the prevailing economic conditions. For example, if the wage rate is lower, the *iso‐profit rate line* is steeper with the origin O=(0,0) as pivot point. This implies that some formerly inferior (superior) methods turn into potential innovations (inventions). See Kurz (2008) for a discussion of invention and innovation in a two‐sector setting.

5 Note that the dynamics of adaptation heavily hinge on the assumption of surplus labour and full utilisation of capacity. The relation between innovation bias and the technology effect is different if labour is inflexibly supplied and full employment prevails (Metcalfe and Steedman, 2013).

6 We here only explore the consequences of firm decline but not the impact of firm exit, which occurs if the invading method lies within the area ΔDAB.
